# Structural and Kinetic Profiling of Rolling Circle Amplification via Solid-State Nanopore Sensing Using miR-21 as a Model

**DOI:** 10.1021/acssensors.5c02039

**Published:** 2025-09-16

**Authors:** Kawin Loha, Thitikorn Boonkoom, Harit Pitakjakpipop, Ibrar Alam, Alongkot Treetong, Poramin Boonbanjong, Itthi Chatnuntawech, Surat Teerapittayanon, Ulrich F. Keyser, Albert Schulte, Deanpen Japrung

**Affiliations:** † School of Biomolecular Science and Engineering (BSE), 423058 Vidyasirimedhi Institute of Science and Technology (VISTEC), Rayong 21210, Thailand; ‡ 61191 National Nanotechnology Center (NANOTEC), National Science and Technology Development Agency (NSTDA), Thailand Science Park, Pathumthani 12120, Thailand; § Program in Translational Medicine, Chakri Naruebodindra Medical Institute, Faculty of Medicine Ramathibodi Hospital, 26687 Mahidol University, Samut Prakan 10540, Thailand; ∥ Cavendish Laboratory, 2152 University of Cambridge, Cambridge CB3 0HE, U.K.

**Keywords:** solid-state nanopore, rolling circle amplification, miR-21, single-molecule analysis, isothermal amplification, nanopore sensing, secondary structure, AFM

## Abstract

Rolling Circle Amplification (RCA) is a robust isothermal nucleic acid amplification technique widely used in molecular diagnostics. In this study, we combine RCA with solid-state nanopore sensing to monitor the amplification process at the single-molecule level using miR-21 as a model biomarker. This label-free platform enables detailed analysis of amplification kinetics and structural transitions over time. Changes in translocation dwell time and current blockage were evaluated across RCA incubation periods (30 min, 1 h, 2 h), revealing time-dependent increases consistent with the generation of longer and more complex DNA concatemers. These findings were validated by Urea-PAGE and atomic force microscopy (AFM), while Mfold-based secondary structure predictions further supported the evolution of more stable and folded configurations. Additionally, a custom-developed signal extraction application facilitated reproducible event classification and visualization. Overall, this integrated approach provides new insights into RCA behavior and highlights the potential of nanopore-based sensing for the development of sensitive, structure-resolved diagnostic tools.

## Graphical abstract

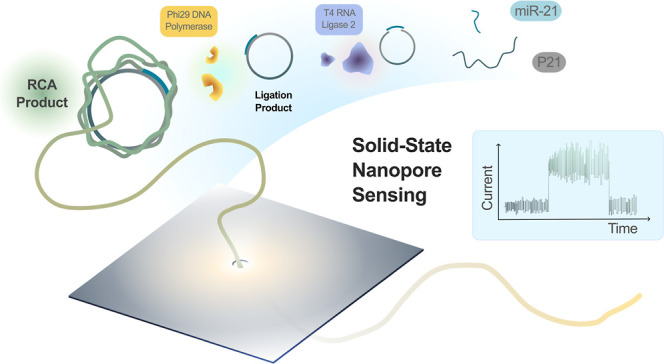

The accurate and sensitive detection of nucleic acids underpins modern molecular diagnostics, offering insights into the genetic and epigenetic basis of diseases ranging from infectious pathogens to cancers. Among emerging biomarkers, microRNAs (miRNAs) such as miR-21 are of particular interest due to their stability in biofluids and key roles in gene regulation, especially in oncogenesis.^1,2^ The detection of miR-21 is therefore pivotal for early disease diagnosis, motivating the development of analytical platforms that are both highly sensitive and capable of single-molecule resolution.

Rolling circle amplification (RCA) has emerged as a robust isothermal amplification strategy well-suited for short nucleic acid targets such as miRNAs. It involves the circularization of a padlock probe followed by isothermal polymerization using Phi29 DNA polymerase, which exhibits high processivity (capable of synthesizing >70 kb products) and a polymerization rate of approximately 50–100 nucleotides per second under standard reaction conditions.^3,4^ These kinetics, applied in this study, enable RCA to generate long single-stranded DNA concatemers with thousands of tandem repeats within 1–2 h, supporting highly sensitive downstream detection.^5^

Conventional RCA detection approaches, such as gel electrophoresis and fluorescence assays, provide only end point readouts and limited structural insight.^6^ Solid-state nanopore technology offers a transformative alternative, enabling label-free, real-time analysis of single nucleic acid molecules.^7^ Nanopores fabricated from materials such as silicon nitride detect transient disruptions in ionic current as biomolecules pass through, providing high sensitivity and the ability to resolve structural features such as length and folding complexity.^8,9^ Recent integrations of RCA with nanopore sensing have demonstrated enhanced detection sensitivity for diverse biomolecules, including nucleic acids and proteins.^10–12^

In this study, we present an integrated RCA—nanopore platform for structural and kinetic profiling of nucleic acid amplification, using miR-21 as a model target (Figure 1). Our approach incorporates padlock probe ligation, Phi29 polymerase-driven RCA, and single-molecule nanopore analysis to monitor translocation dynamics across increasing amplification durations. By leveraging the known synthesis kinetics of Phi29, we estimate concatemer length and repeat number over time. The morphological evolution of RCA products is corroborated by atomic force microscopy (AFM) and theoretical secondary structure predictions. To support efficient signal analysis, we also developed a custom web-based event extraction tool for imaging and classifying translocation events. Together, this approach enables detailed, label-free monitoring of amplification behavior at the single-molecule level and highlights the potential of nanopore platforms for sensitive and structure-informed molecular diagnostics.

**Figure 1:**
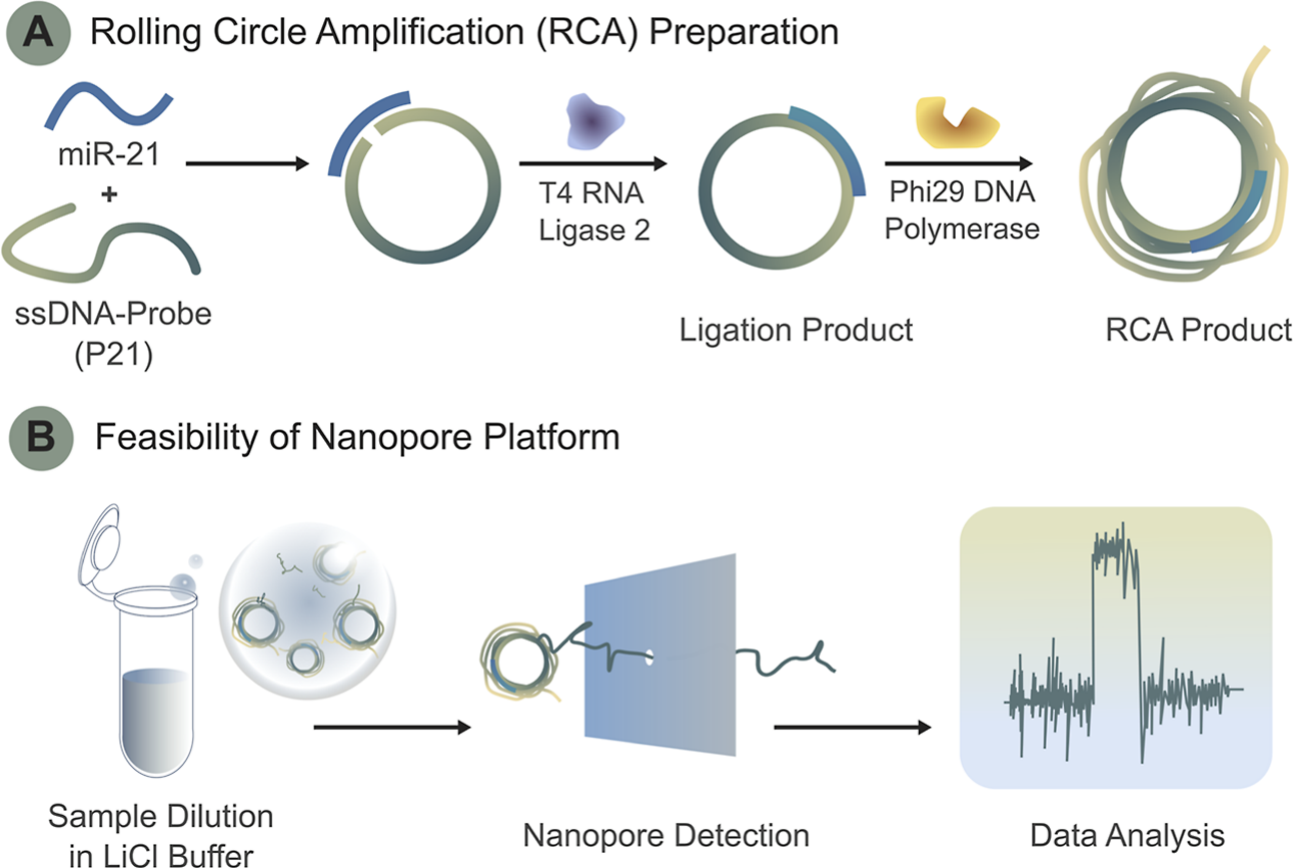
Schematic overview of the rolling circle amplification (RCA) assay for miR-21 monitored using nanopore technology. (A) Illustration of the RCA process, showing miR-21 hybridization with the padlock probe (P21), ligation by T4 RNA ligase 2, and amplification by Phi29 DNA polymerase to generate long concatemeric products. (B) Overview of solid-state nanopore analysis, depicting sample dilution, translocation through the nanopore, and ionic current signal analysis for single-molecule detection.

## Materials and Methods

### Materials

Linear padlock DNA probes (P21) and synthetic miR-21 oligonucleotides were purchased from Integrated DNA Technologies (IDT). T4 RNA ligase 2 (T4 Rnl2), Phi29 DNA polymerase, and associated buffers were obtained from New England Biolabs (NEB). SYBR Gold nucleic acid stain was sourced from Thermo Fisher Scientific. Solid-state silicon nitride (SiN) membranes were provided by Norcada. Silver wires (1 mm, 99.9% purity), lithium chloride (LiCl), potassium chloride (KCl), and all other reagents were analytical grade. The P21 padlock probe and miR-21 target sequences as shown in Table 1, were adopted from our previous work.^13,14^

**Table 1:** Oligonucleotide Sequences Used in This Study^a^

oligonucleotide	sequences (5′ → 3′)
P21 probe (76 nt)	PO_4_ C̲T̲G̲ A̲T̲A̲ A̲G̲C̲ T̲A̲C AAC CAA AAC CAA CAC CAA TTC CAC ACC TCC CTA CAC AAC CAA AAC CAA CAC CAT̲ C̲A̲ A̲C̲A̲ T̲C̲A̲ G̲T̲
miR-21 (22 nt)	UAG CUU AUC AG ACU GAU GUU GA

a The underlined bases in the P21 probe indicate the miRNA binding sites.

### RCA Reaction

#### Ligation Reaction

To generate circular DNA probes, a 10 μL ligation reaction mixture (10 μL) was prepared containing 1 μL of 100 nM linear DNA probe (P21), 1 μL of 10 nM miR-21, 1 μL of 10X ligation buffer, 1 μL (10 units) of T4 Rnl2, and 6 μL of RNase-free water. A mixture of P21, miR-21 and water was first heated at 65 °C for 3 min, gradually cooled for 10 min. The T4 Rnl2 and ligation buffer were then added and incubated for 1 h at 37 °C. For nanopore experiments, the ligation product was stored at −20 °C for downstream analysis.

#### Amplification Reaction

The RCA mixture (20 μL) contained the ligated product (10 μL), 2 μL of 2 mg/mL BSA, 2 μL of 10 mM dNTPs, 2 μL of 10X reaction buffer, 10 U of Phi29 DNA polymerase, and sterile water. Incubation was carried out at 37 °C for 30 min, 1 h, and 2 h, followed by enzyme inactivation at 65 °C for 10 min. The amplified products were then stored at −20 °C.

#### Gel Electrophoresis (Urea-PAGE)

To confirm ligation and amplification, samples were analyzed on 10% Urea-PAGE in 1X TBE at 200 V for 40 min. Gels were stained with SYBR Gold for 30 min and imaged using the In-Vivo FX PRO system.

### Nanopore Device Fabrication and Conditioning

Solid-state nanopores were fabricated using the dielectric breakdown method^15^ with the Northern Nanopore Instrument Spark-E2 (version 8.0.0). Prior to fabrication, silicon nitride (SiN) membranes were thoroughly cleaned with 2-propanol to remove organic contaminants. The cleaned membranes were mounted into a flow cell assembly using appropriate gaskets to ensure a leak-tight seal. To enhance membrane surface hydrophilicity and remove residual contaminants, both cis and trans chambers of the flow cell were sequentially flushed with 80 μL of 2-propanol, followed by sterile water, and finally with 80 μL of degassed 1 M KCl containing 10 mM HEPES (pH 8.0).

Ag/AgCl electrodes (1 mm diameter) were prepared by immersing silver wires in 5% sodium hypochlorite (NaClO) for at least 30 min. Electrodes were then inserted into antievaporation caps and placed into each chamber of the flow cell. Care was taken to avoid overfilling during assembly to prevent electrode corrosion or flow cell damage. The assembled flow cell was loaded into the Spark-E2 instrument, and the standard dielectric breakdown protocol was initiated. The system was calibrated using the known conductivities of 1 M KCl (10.6 ± 0.5 S/m) and 3.6 M LiCl (16.5 ± 0.5 S/m), both containing 10 mM HEPES (pH 8.0). An increasing voltage was applied across the membrane until the formation of a nanoscopic defect was detected by a sharp increase in current, indicating successful pore formation.

Following breakdown, the buffer was replaced with 3.6 M LiCl (10 mM HEPES, pH 8.0) to initiate the conditioning step, which promotes pore expansion and stabilization. The system continued conditioning until the nanopore reached the desired diameter of approximately 8 nm, at which point the process automatically terminated. The pore was then ready for subsequent translocation experiments. This fabrication and conditioning protocol closely followed procedures established in our previous work.^16^

### Nanopore Measurements

Nanopore experiments were performed using an Axopatch 200B patch clamp instrument (Molecular Devices, USA) connected to two Ag/AgCl electrodes and a flow cell containing the prepared nanopore, as described above. All measurements were conducted with the “cis” chamber grounded, and sterile 3.6 M LiCl containing 10 mM HEPES (pH 8.0) was used as the running buffer. For single molecule detection, each sample was diluted 25-fold in LiCl buffer and added to the “trans” side of the flow cell. A negative voltage was applied to drive negatively charged species through the nanopore. The ionic current was recorded at a sampling rate of 50 kHz and filtered at 10 kHz. Single molecule translocation events were analyzed using Clampfit 11.2 and OriginPro (version 2021b). Data analysis included Gaussian fitting to determine the percentage of current blockage and exponential fitting for dwell time estimation, along with graphical display of the results.

### Application Development for Nanopore Event Extraction Web Application

To enable more detailed and reproducible analysis of nanopore translocation signals, we developed a custom graphical user interface (GUI)-based application (Figure S1). This application allows users to adjust key parameters, including the baseline current level (lvl0), event detection threshold (lvl1), baseline tolerance, and visualization padding. The baseline level (lvl0) defines the open-pore current under stable conditions, while the threshold (lvl1) specifies the minimum current deviation required to trigger event detection. Baseline tolerance accounts for minor signal fluctuations, preventing false-positive detections from noise. The padding parameter allows additional data points before and after each detected event to be captured for full signal context. Events are automatically identified when the current crosses the specified threshold, and extracted traces are analyzed to calculate key parameters such as dwell time and percentage current blockage.

### Secondary Structure Prediction

To investigate the conformational behavior of the ligated circular probe and RCA products, secondary structure predictions were performed using the Mfold web server.^17^ All sequences were analyzed at 25 °C using a sodium ion concentration of 3 M to match the ionic strength of the nanopore sensing buffer. For the circular probe (P21), the structure was computed using Mfold’s circular DNA option. For RCA products, a 2000-nucleotide sequence composed of tandem repeats of the reverse complement of P21 was used as a representative model due to software length limitations. The predicted structures were evaluated based on Gibbs free energy (Δ*G*) and the presence of repetitive structural motifs, such as stem–loops, to correlate with the observed nanopore translocation signatures and AFM results.

### Atomic Force Microscopy

RCA products were deposited on freshly cleaved mica, air-dried, and imaged using NanoWizard 3 (JPK Instruments) in AC mode. Olympus OMCL-AC200TS tips were used (resonance frequency 150 kHz, spring constant 9 N/m). Images were acquired at 256 × 256 pixels and 1 Hz scan rate.

## Results and Discussion

### PAGE Validation of RCA Products

The principle of Rolling Circle Amplification (RCA) for miR-21 detection is illustrated in Figure 1A. A single-stranded DNA padlock probe (P21) was designed with complementary sequences at its 5′ and 3′ termini, allowing hybridization to adjacent regions of the target miR-21. Upon hybridization, the ends of the probe were ligated by T4 RNA ligase 2 (T4 Rnl2) to form a circular DNA template through phosphodiester bond formation. In this configuration, miR-21 also functions as the primer, enabling Phi29 DNA polymerase to initiate amplification and generate long concatemeric single-stranded DNA consisting of repeated complementary sequences of the probe.

The success of the ligation and amplification steps was initially assessed using Urea-PAGE analysis (Figure 2). A distinct shift in band position after ligation (lane 5) confirmed the formation of the circularized probe–miR-21 complex. Following RCA, the products remained near the wells (lanes 7–9), consistent with the formation of high-molecular-weight DNA across all incubation times tested (30 min, 1 h, and 2 h).

**Figure 2:**
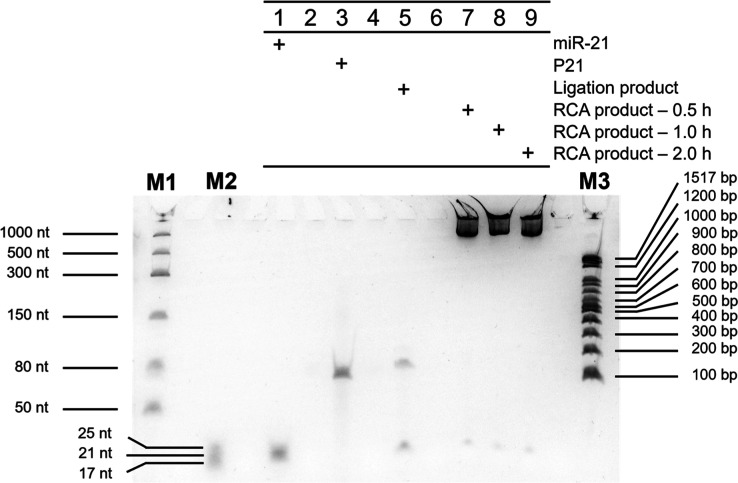
Urea-PAGE (10%) analysis of miR-21 ligation and RCA products over time. Lanes M1 and M2 contain a low-range ssRNA ladder and a synthetic miRNA marker, respectively. Lane 1 shows the migration of free miR-21, while lane 3 contains the linear single-stranded DNA padlock probe (P21). Lane 5 displays the ligation product of P21 with miR-21 in the presence of T4 RNA ligase 2 (T4 Rnl2), indicating the formation of a circular DNA–RNA hybrid complex. Lanes 7, 8, and 9 correspond to rolling circle amplification (RCA) products after 0.5, 1.0, and 2.0 h of incubation, respectively, all showing strong retention near the wells consistent with high molecular weight concatemer formation. Lane M3 is a 100 bp DNA ladder used as a reference for larger product sizes.

A faint residual band corresponding to miR-21 was still visible after the ligation step, which may result from either a small portion of unligated target due to incomplete hybridization or from partial dissociation of the circular probe–miR-21 complex under the denaturing conditions of Urea-PAGE, as short RNA–DNA hybrids can be disrupted by urea and applied voltage.^18^ In either case, this observation does not indicate inefficient ligation, as the linear probe was no longer detectable and robust amplification was consistently observed.

To investigate the progression of amplification over time, RCA products were analyzed after 30 min, 1 h, and 2 h of incubation. The observed increase in product size with longer incubation times is consistent with the high processivity and strand displacement activity of Phi29 DNA polymerase, which is capable of synthesizing DNA at an average rate of 50–100 nucleotides per second under optimal conditions.^3,4^ Given a circular template of ∼76 nucleotides, each complete replication round adds an equivalent-length repeat to the growing strand. At this rate, a single RCA reaction could generate approximately 90,000–180,000 nucleotides within 30 min, 180,000–360,000 nt in 1 h, and 360,000–720,000 nt in 2 h of continuous amplification. This high product yield is further supported by the exceptional processivity of Phi29 polymerase, which has been reported to synthesize tens to hundreds of kilobases without dissociation from the template. The enzyme’s ability to remain stably bound and active for extended periods under isothermal conditions makes it particularly well-suited for RCA and contributes to the formation of ultralong concatemeric products.

The accumulation of these long single-stranded DNA products was reflected in Urea-PAGE analysis. For each time point, the RCA products were largely retained near the wells of the gel and did not migrate appreciably. This behavior is attributed to the high molecular weight and increased negative charge density of the concatemeric products, which limit their mobility through the polyacrylamide matrix. Additionally, denaturing conditions (8 M urea) and the high voltage typically used in PAGE (e.g., 200 V) may further affect the structural flexibility of long single-stranded DNA, leading to partial folding or entanglement that further impedes gel migration. While Urea-PAGE did not reveal a clearly increasing band intensity across time points, the persistent well retention of RCA products at all incubation durations indicates the formation of high-molecular-weight concatemers. This observation is consistent with the known processivity and polymerization kinetics of Phi29 DNA polymerase under isothermal conditions.^3,4^

These PAGE results therefore confirm the time-dependent elongation of RCA products and provide a robust biochemical foundation for subsequent single-molecule analysis using nanopore sensing.

### Nanopore Fabrication, Characterization and Initial Observations

To obtain single-molecule information beyond the ensemble trends by PAGE, we next fabricated and characterized solid-state nanopores for the analysis of RCA products. Solid-state silicon nitride (SiN) nanopore was fabricated using the dielectric breakdown method, in which a strong electric field is applied across a thin membrane to induce pore formation. Once the membrane reaches a threshold current, a sudden increase in ionic current signals the formation of a nanoscale defect, triggering the conditioning process to fine-tune the nanopore diameter.

Figure 3A illustrates the step-by-step breakdown process during pore fabrication in 1 M KCl containing 10 mM HEPES (pH 8). The negative applied voltage was gradually increased in a staircase fashion, and the current was monitored simultaneously. An initial stable low-level leakage current was observed until a sharp rise to approximately 150 nA indicated successful dielectric breakdown in the 12 ± 2 nm-thick SiN membrane. This threshold is consistent with prior reports for nanopore formation in membranes of similar thickness and under comparable field strengths.^19^

**Figure 3:**
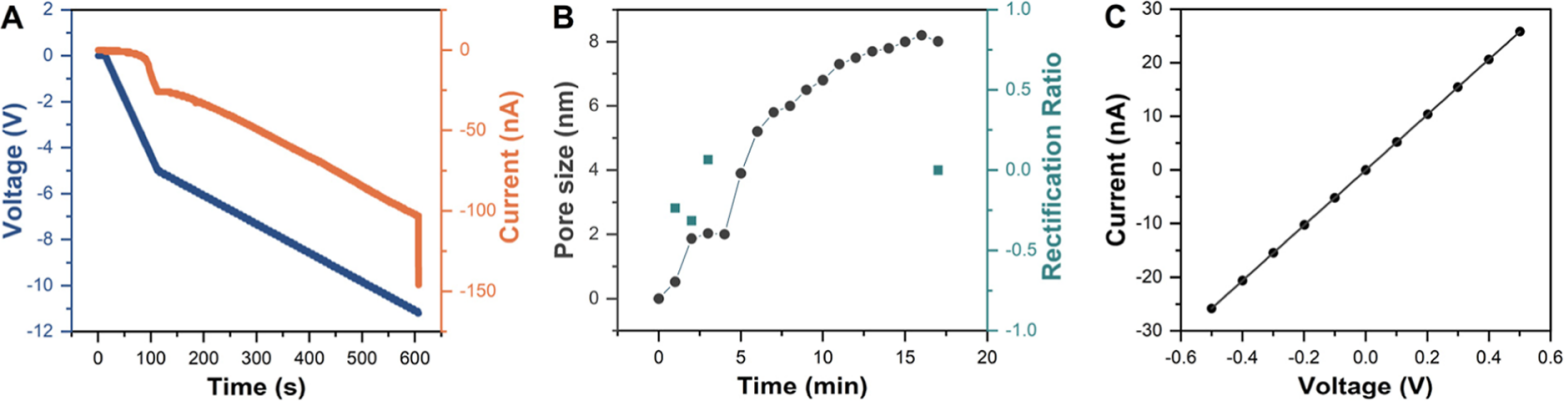
Solid-state nanopore fabrication and electrical characterization. (A) Time-dependent voltage and current profile during dielectric breakdown-based nanopore fabrication. The negative applied voltage was gradually increased in a staircase fashion across a 12 ± 2 nm-thick SiN membrane immersed in 1 M KCl containing 10 mM HEPES (pH 8). A sharp increase in current (∼150 nA) indicates successful breakdown and nanopore formation. (B) Conditioning step performed in 3.6 M LiCl with 10 mM HEPES (pH 8), showing the enlargement of pore size over time and a decreasing rectification ratio approaching zero, consistent with symmetric pore geometry. (C) Current–voltage (I–V) curve of the fabricated nanopore under symmetric ionic conditions (3.6 M LiCl), showing linear and ohmic behavior, confirming pore stability and the absence of significant rectification.

After defect formation, the solution was replaced with 3.6 M LiCl containing 10 mM HEPES (pH 8) to initiate the conditioning step, allowing the pore to gradually enlarge. All subsequent nanopore measurements were carried out in this buffer. LiCl was chosen because it slows DNA translocation and improves signal-to-noise ratios, enabling better resolution of long RCA products. Mg^2+^, while required during the RCA reaction itself, was excluded from sensing conditions because divalent cations can promote DNA condensation and nanopore clogging, reducing reproducibility. As shown in Figure 3B, the pore size increased over time and stabilized at approximately 8 nm after ∼15 min. Simultaneously, the rectification ratio approached zero, suggesting a symmetric and geometrically stable pore, suitable for conductance-based sizing and single-molecule sensing.

#### Note on Imaging Limitation

Due to the random nature of nanopore formation using the dielectric breakdown method across the 40 × 40 μm SiN diaphragm, direct visualization of the specific pore using high-resolution TEM is technically challenging. As a result, pore size estimation in this study was based on conductance measurements—a method widely accepted and validated within the nanopore research community. Previous studies have demonstrated that conductance-based sizing provides reliable diameter estimates consistent with high-resolution imaging, particularly in dielectric breakdown-fabricated nanopores and in long-term stability studies using similar platforms.^15^ Future work may explore integrating fabrication approaches that are more compatible with direct imaging to complement electrical characterization.

To verify the pore’s geometry and electrical behavior, we recorded its current–voltage (I–V) characteristics under symmetric salt conditions (3.6 M LiCl) (Figure 3C). The resulting linear I–V response confirmed ohmic behavior and the absence of significant current rectification, further supporting the formation of a symmetric pore. Additionally, the low baseline noise indicated a clean nanopore with good sealing and minimal surface defects.

The pore conductance (*G*) was obtained from the slope of the I–V curve and used to estimate nanopore diameter using the following commonly accepted equation^20^

$$G=\sigma {\left[\frac{4L}{\pi d^{2}}+\frac{1}{d}\right]}^{-1}$$

where *G* is the nanopore conductance (S), σ is the bulk ionic conductivity (16.5 ± 0.5 S/m for 3.6 M LiCl), *L* is the membrane thickness (12 nm ± 2 nm), and *d* is the nanopore diameter. This model assumes a cylindrical pore geometry and excludes surface charge effects. Conductance-based estimation confirmed the formation of an ∼8 nm nanopore, whose stability and reliability make it suitable for single-molecule RCA analysis. In the following section, we employ this platform to investigate how RCA products of varying amplification times translocate through the nanopore.

### Nanopore Analysis of Ligation Product

To validate the molecular identity of the ligation product prior to RCA, we performed single-molecule nanopore sensing using a previously characterized ∼8 nm solid-state SiN nanopore. Four experimental conditions were tested: (i) pure buffer (3.6 M LiCl with 10 mM HEPES, pH 8); (ii) a mixture of padlock probe (P21) (1 μL of 100 nM) and miR-21 (1 μL of 10 nM); (iii) T4 RNA ligase 2 alone (1 μL of 10 U); and (iv) the full ligation reaction mixture, consisting of 1 μL of 100 nM linear DNA probe (P21), 1 μL of 10 nM miR-21, 1 μL of 10X ligation buffer, 1 μL (10 units) of T4 Rnl2, and 6 μL of RNase-free water. All analyte-containing samples were diluted 25-fold into LiCl buffer, vortexed briefly, and introduced into the trans chamber of the flow cell; the cis side contained only buffer. A voltage of −300 mV was applied to drive negatively charged species through the nanopore.

After dilution, the estimated final concentration of the ligation product was 0.4 nM, which falls within the typical detection range for single-molecule nanopore sensing (see Supporting Information Note for calculation of both ligation product and enzyme concentrations). As shown in Figure 4A, no translocation events were observed in the buffer-only, P21/miR-21 mixture, or enzyme-only control, confirming that the recorded signals are specific to the ligation product. Notably, the absence of signal in the enzyme-only control is consistent with the low estimated concentration of T4 RNA ligase 2 (∼6 nM, based on 10 U in 10 μL diluted 25-fold; see Supporting Information Note), and its near-neutral or slightly positive charge at pH 8, which reduces its electrophoretic mobility under the applied negative voltage during nanopore sensing.

**Figure 4:**
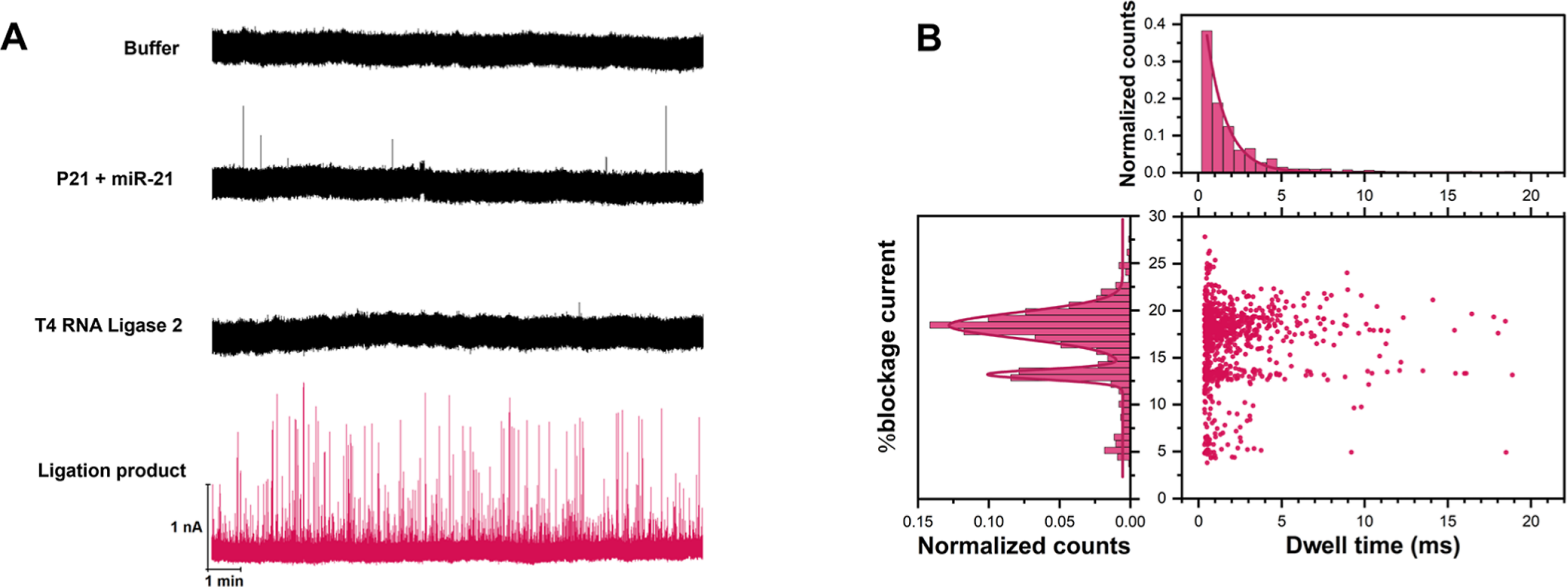
Single-molecule nanopore sensing of ligation product using an 8 nm solid-state SiN nanopore. (A) Representative current traces of four sample conditions: pure buffer (top), P21 + miR-21, T4 Rnl2 alone, and the full ligation product (bottom, red trace). Only the ligation product generates frequent translocation events. (B) Scatter plot of %blockage current versus dwell time for the ligation product, with accompanying histograms. Two distinct populations are identified via Gaussian fitting, potentially corresponding to circular P21 with and without miR-21 hybridization.

While it is theoretically possible that the ligation product forms transient complexes with the enzyme, such interactions are unlikely under our experimental conditions. The 3.6 M LiCl buffer is known to weaken protein–nucleic acid interactions and promote dissociation of loosely bound complexes.^21^ Therefore, it is expected that T4 RNA ligase 2 dissociated from the circularized DNA before or during translocation.

Figure 4B presents the scatter plot of % blockage current versus dwell time for the ligation product, along with corresponding histograms. Two distinct populations were identified by Gaussian fitting, centered at 13.2% ± 0.04 and 18.4% ± 0.05 blockage. These populations are potentially attributable to structural differences: the lower % blockage signal (13.2%) is accompanied by shorter dwell times (∼0.6 ms), suggesting rapid translocation of a more compact and rigid structure—likely circular P21 hybridized with miR-21, forming a partial duplex. In contrast, the higher % blockage population (18.4%) exhibits longer and more variable dwell times (∼1.2–2.0 ms), consistent with unhybridized circular P21 adopting flexible, heterogeneous conformations that result in deeper and slower nanopore interactions. These trends are consistent with prior studies showing that ssDNA or circular DNA with greater conformational freedom translocates more slowly and with deeper blockage signals in ∼8 nm solid-state nanopores.^22,23^

Theoretical structure prediction using Mfold supports this interpretation, revealing that unhybridized P21 adopts multiple low-stability conformations (Supporting Information Figure S2), while hybridization with miR-21 stabilizes the loop region, creating a more defined structure as illustrated in Figure 5.

**Figure 5:**
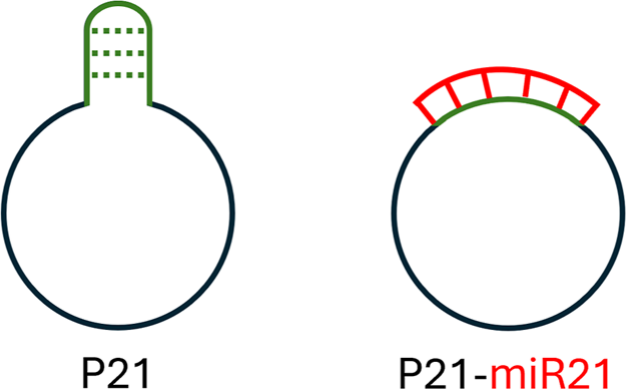
Schematic illustration of the predicted structural differences between the circular DNA probe P21 alone (left) and hybridized with miR-21 (right). In the unbound state, P21 adopts a flexible conformation with minimal secondary structure (shown in green). Upon hybridization with miR-21 (shown in red), the loop region becomes stabilized through duplex formation, resulting in a more defined and rigid structure. This depiction is based on theoretical secondary structure predictions using the Mfold server and supports the observed nanopore translocation behavior under high-salt conditions (3.6 M LiCl, −300 mV).

Importantly, the hybridization between miR-21 and the loop region of P21 is expected to remain intact during nanopore analysis. Although 3.6 M LiCl provides slightly less stabilization for DNA–RNA duplexes compared to NaCl or KCl, the 22 nt duplex formed between miR-21 and P21 remains thermodynamically stable. Furthermore, while the applied voltage of −300 mV imposes an electrophoretic force, the stability of the duplex is sufficient to withstand disruption under these conditions. This interpretation is consistent with the observed translocation signals and further supported by the structural models presented in the Supporting Information Together, these results establish a validated baseline for subsequent investigation of RCA products at different amplification times.

### RCA Time-dependent Translocation Behavior

To monitor the progression of the RCA reaction, we analyzed the translocation behavior of the resulting DNA concatemers using single molecule nanopore sensing. RCA reactions were performed for three different incubation times: 30 min, 1 h, and 2 h. After amplification, the reaction products were diluted 25-fold into 3.6 M LiCl containing 10 mM HEPES (pH 8) and added to the trans chamber of the flow cell. The cis chamber contained only LiCl buffer, and a constant voltage of −300 mV was applied.

Representative ionic current traces of the three RCA samples are shown in Figure 6A–C. The corresponding translocation data, scatter plots of % blockage current versus dwell time with accompanying histograms, are shown in Figure 6D–F. Each data set was fitted with a Gaussian distribution to characterize % blockage current and exponential fit for dwell time.

**Figure 6:**
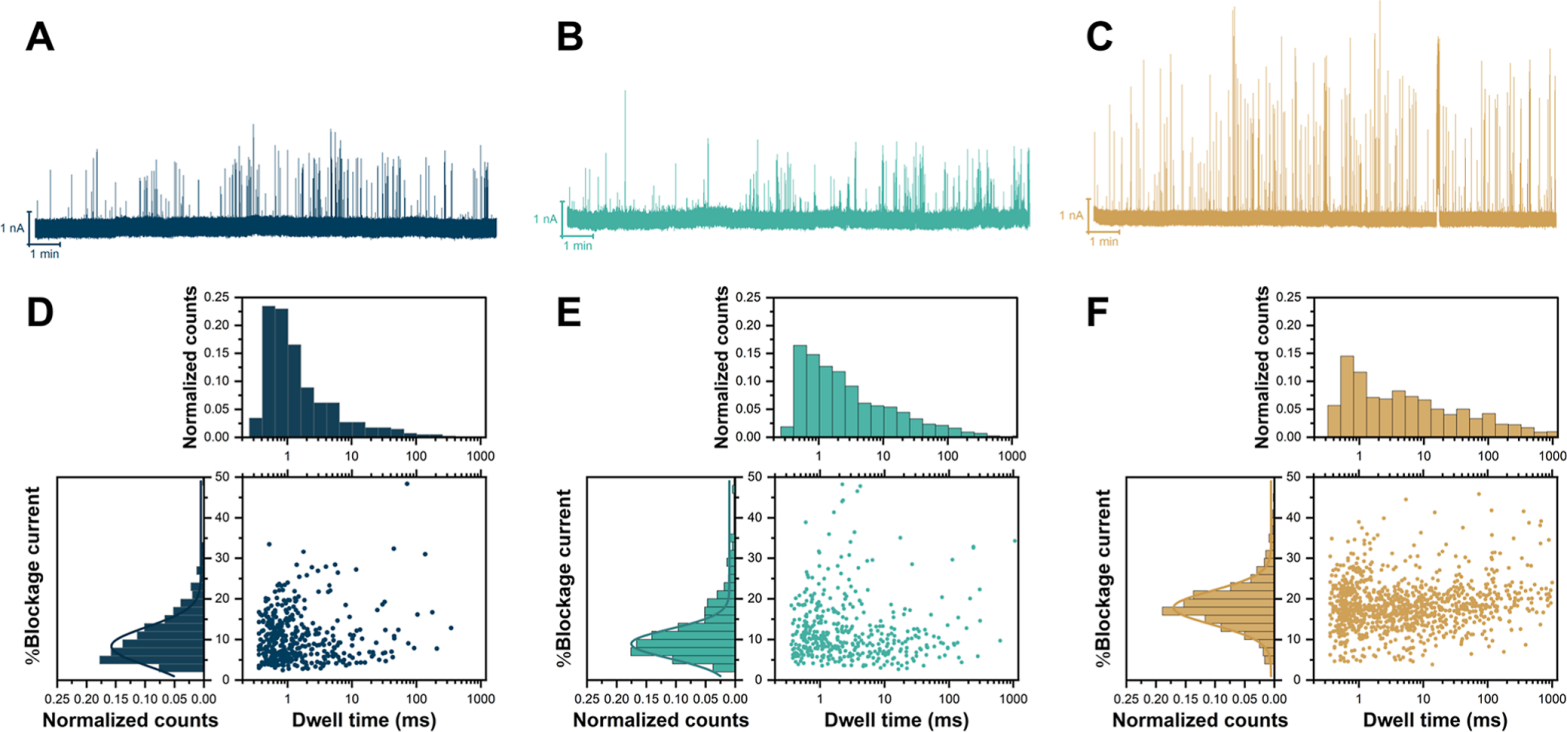
Nanopore sensing of RCA products at different incubation times using an 8 nm solid-state silicon nitride (SiN) nanopore. (A–C) Representative ionic current traces for RCA products after 30 min (A), 1 h (B), and 2 h (C) of amplification. A higher frequency of translocation events and deeper current blockages are observed with longer incubation times. (D–F) Scatter plots of percentage current blockage versus dwell time for each corresponding incubation period, accompanied by histograms. Both current blockage and dwell time distributions were fitted with Gaussian and exponential function. The progressive increase in dwell time and percentage current blockage with amplification time is consistent with the formation of longer and more structurally complex RCA products.

The results indicate that both dwell time and % blockage current increased with longer incubation times, consistent with the progressive elongation and structural complexity of RCA products. As detailed earlier, Phi29 polymerase produces long concatemeric strands by continuously appending repeats of the circular template. Given the 76 nt length of the P21 template, this corresponds to approximately 1200–9500 tandem repeats per concatemer, depending on reaction duration and enzyme performance.

To better understand the structural complexity of the RCA products and its potential impact on nanopore translocation behavior, we predicted the secondary structures of a 2000-nucleotide RCA concatemer sequence using the Mfold web server under experimental conditions (3 M Na^+^, 25 °C). Although the theoretical length of RCA products in our experiments may reach hundreds of kilobases, we were limited by Mfold’s input constraints and therefore modeled a 2000 nt fragment as a representative segment. This allowed us to capture key structural motifs such as repetitive stem–loop domains, which are likely to be repeated and amplified in longer RCA concatemers. The predicted structure, consisting of ∼26 tandem repeats of the 76 nt reverse complement of the P21 probe, revealed 13 distinct stem–loop motifs distributed along the sequence. The Gibbs free energy of this structure was calculated to be −104.32 kcal/mol, indicating high thermodynamic stability even in high-salt conditions. Given that actual RCA products from 1–2 h reactions are likely tens or hundreds of kilobases in length, they may contain dozens to hundreds of such folded subdomains, resulting in complex, modular topologies. These structural features help explain the increases in dwell time and current blockage observed in nanopore measurements as incubation time increases. Figure 7 presents the predicted secondary structure of this 2000 nt model concatemer, illustrating the repetitive and highly structured nature of the amplified DNA.

**Figure 7:**
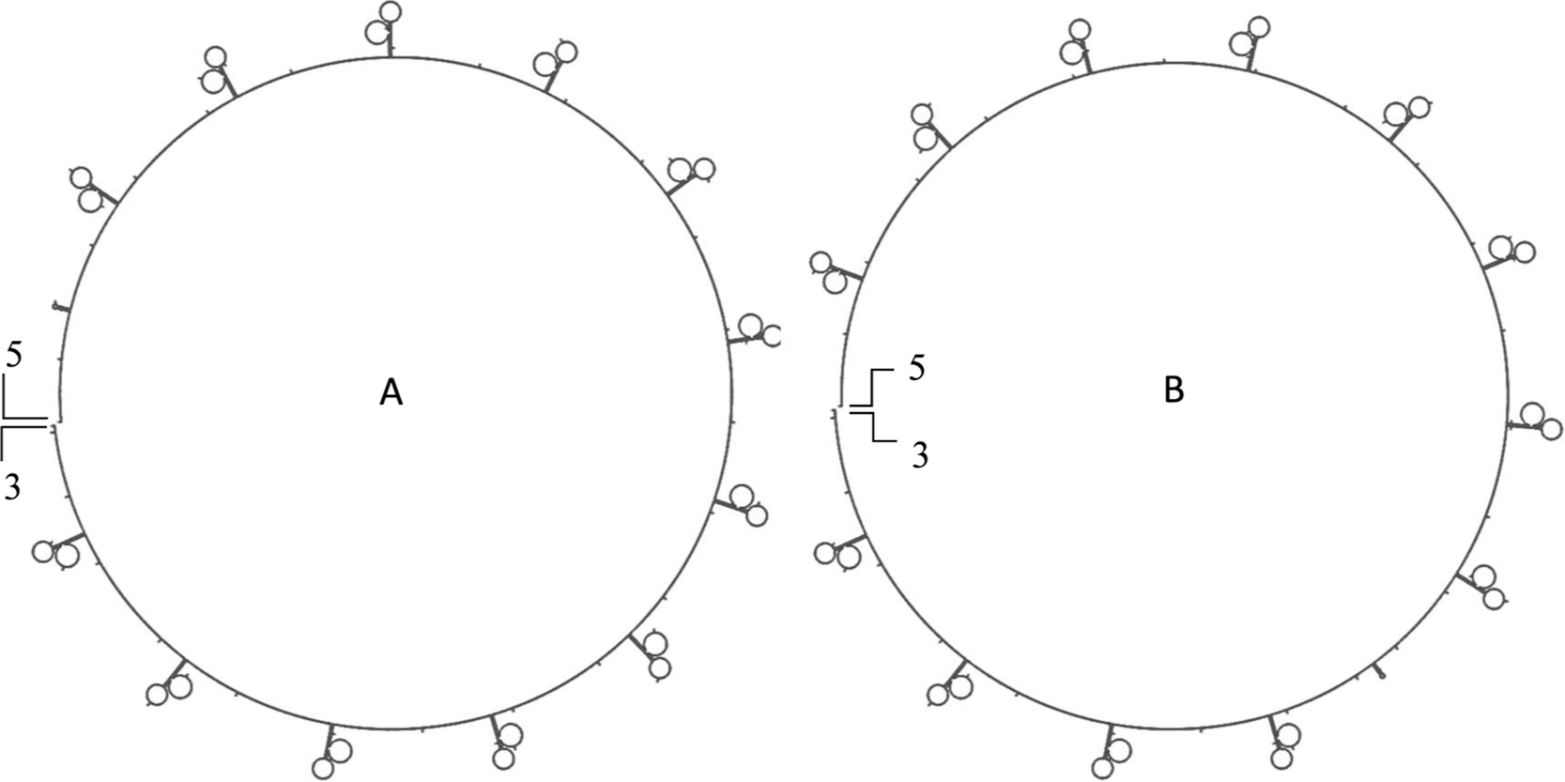
Predicted secondary structures of the linear rolling circle amplification (RCA) product containing 2000 nucleotides, generated from the reverse complement of the circular P21 probe. (A,B) show two representative low-energy conformations predicted using the Mfold web server^17^ under conditions simulating the nanopore analysis environment (3 M Na^+^, 25 °C). The RCA product consists of ∼26 tandem repeats of the 76-nucleotide reverse complement of P21, continuously synthesized by Phi29 DNA polymerase. Both structures exhibit multiple recurring stem–loop motifs corresponding to individual repeat units. A small gap is included to indicate the linear nature of the sequence, with the 5′ end labeled as the initiation point. The predicted Gibbs free energy for both structures is Δ*G* = −104.32 kcal/mol, indicating highly stable secondary structure formation under these conditions.

To ensure that the recorded nanopore signals originated from RCA products rather than other reaction components, we considered potential contributions from proteins present in the amplification mixture, including T4 RNA ligase 2, Phi29 DNA polymerase, and bovine serum albumin (BSA). Control experiments shown in Figure 4 demonstrate that T4 RNA ligase 2 alone does not produce detectable translocation signals, consistent with its low concentration (∼6 nM) and limited electrophoretic mobility under the applied −300 mV bias. While dedicated control experiments were not performed for Phi29 polymerase or BSA, their estimated concentrations after dilution were low (∼15.7 nM for Phi29 and ∼60.8 nM for BSA; see Supporting Information for calculation details). Both proteins are globular and possess near-neutral or slightly positive surface charges at pH 8. In particular, BSA exhibits reduced zeta potential under high-salt conditions, diminishing its effective charge and mobility under electrophoretic driving forces. This observation is consistent with earlier studies on protein dynamics in high ionic strength conditions, which demonstrate reduced protein activity and nucleic acid structural alterations under such environments.^24,25^ Together, these findings reinforce the interpretation that the signals detected in nanopore analysis predominantly originate from DNA-based RCA products rather than from protein contaminants.

Statistical analysis (Table 2) highlights a substantial increase in mean dwell time from the ligation product (1.18 ms) to RCA-2 h (88.3 ms), indicating the formation of significantly longer DNA concatemers over time. Furthermore, shifts in % blockage current also allow differentiation of products at various amplification stages, demonstrating that nanopore sensing can successfully monitor RCA progression at the single-molecule level.

**Table 2:** Statistical Characteristics of the Mean Peak % Blockage Current and Dwell Time for the Ligation Product and RCA Products at Different Time Points^a^

sample	%blockade current	dwell time (ms)
ligation product	13.2 ± 0.1 and 18.4 ± 0.1	1.2 ± 0.1
RCA-0.5 h	8.3 ± 0.4	4.7 ± 2.4
RCA-1 h	8.9 ± 0.3	19.2 ± 8.3
RCA-2 h	17.8 ± 0.2	88.3 ± 30.4

a Error values (±) represent standard deviations derived from Gaussian and exponential fitting of the translocation event distributions.

It is worth noting that the ligation product displayed a higher mean % blockade current than the RCA-0.5 h and RCA-1 h products (Table 2). While this might appear counterintuitive given the expected increase in molecular length during RCA, this trend can be explained by structural differences. The ligation product, particularly in its circularized and partially duplexed form, adopts compact and relatively rigid conformations (Figure 5, Supporting Information Figure S2), which tend to generate transient but deeper blockades upon translocation through the ∼8 nm nanopore. In contrast, the early RCA products form longer, more flexible concatemeric strands that thread progressively through the pore, leading to shallower mean blockades but significantly longer dwell times. This behavior highlights that blockade current is influenced not only by molecular size but also by compactness, rigidity, and folding state, consistent with previous reports of nanopore sensing of ssDNA and concatemeric structures.

Beyond product tracking, this approach provides valuable mechanistic insights into the RCA reaction itself. The progressive increase in signal complexity with incubation time reflects the stochastic yet processive behavior of Phi29 DNA polymerase, which generates increasingly long concatemeric products from circular templates. Mfold-based structure predictions support the formation of repetitive stem–loop domains that contribute to the observed increase in translocation dwell time and blockage current. These features suggest that the RCA products not only increase in size, but also fold into increasingly complex and structured configurations over time. As such, nanopore analysis enables direct, label-free observation of structural evolution during RCA and serves as a useful tool for probing the kinetics, uniformity, and conformational behavior of DNA amplification reactions at the single-molecule level. In this study, nanopores with an effective diameter of ∼8 nm were chosen for RCA product analysis. Although smaller pores of ∼6 nm have been used in some prior studies to probe short nucleic acids and compact duplexes, we found that such pores tend to clog frequently when challenged with the long concatemeric products generated during RCA. In contrast, larger pores (>10 nm) often produce shallow blockades, making it difficult to discriminate between structural states. The ∼8 nm size therefore represents a practical compromise, providing sufficient sensitivity to resolve structural differences while maintaining stability during long recordings. This choice is also consistent with previous reports demonstrating the suitability of ∼8 nm pores for single-molecule analysis of circular and linear nucleic acids fabricated by dielectric breakdown. While the present work focused on proof-of-concept RCA monitoring using a single pore size, future studies will systematically investigate multiple nanopore diameters to further validate the robustness and generality of the observed trends.

### Classification and Interpretation of Nanopore Translocation Events

To further investigate the structural dynamics of RCA products during nanopore sensing, a custom-developed web-based graphical user interface (GUI) was used to extract and analyze individual translocation events. Raw ionic current traces were uploaded, and baseline (lvl0), threshold (lvl1), tolerance, and padding parameters were adjusted to define event boundaries. Events were detected when the current deviated beyond the set threshold, and each trace was analyzed to determine dwell time and percentage current blockage. This approach enabled consistent, parameter-defined extraction of event data across different RCA conditions, with manual validation performed to ensure reliability.

Extracted events were classified into three dwell time categories: short (<0.3 ms), medium (0.3–1.0 ms), and long (>1.0 ms) (Figure 8). Representative current traces illustrated each category, with all three types contributing approximately equally to the total event population. Short events, predominantly found in the 30 min RCA sample, were attributed to short or minimally folded DNA products. Medium events reflected partially structured concatemers, while long dwell time events corresponded to highly folded or entangled RCA products. The increasing proportion of long events—from 9% at 30 min to 14% at 1 h and 20% at 2 h—supports the time-dependent structural growth and complexity of RCA molecules.

**Figure 8:**
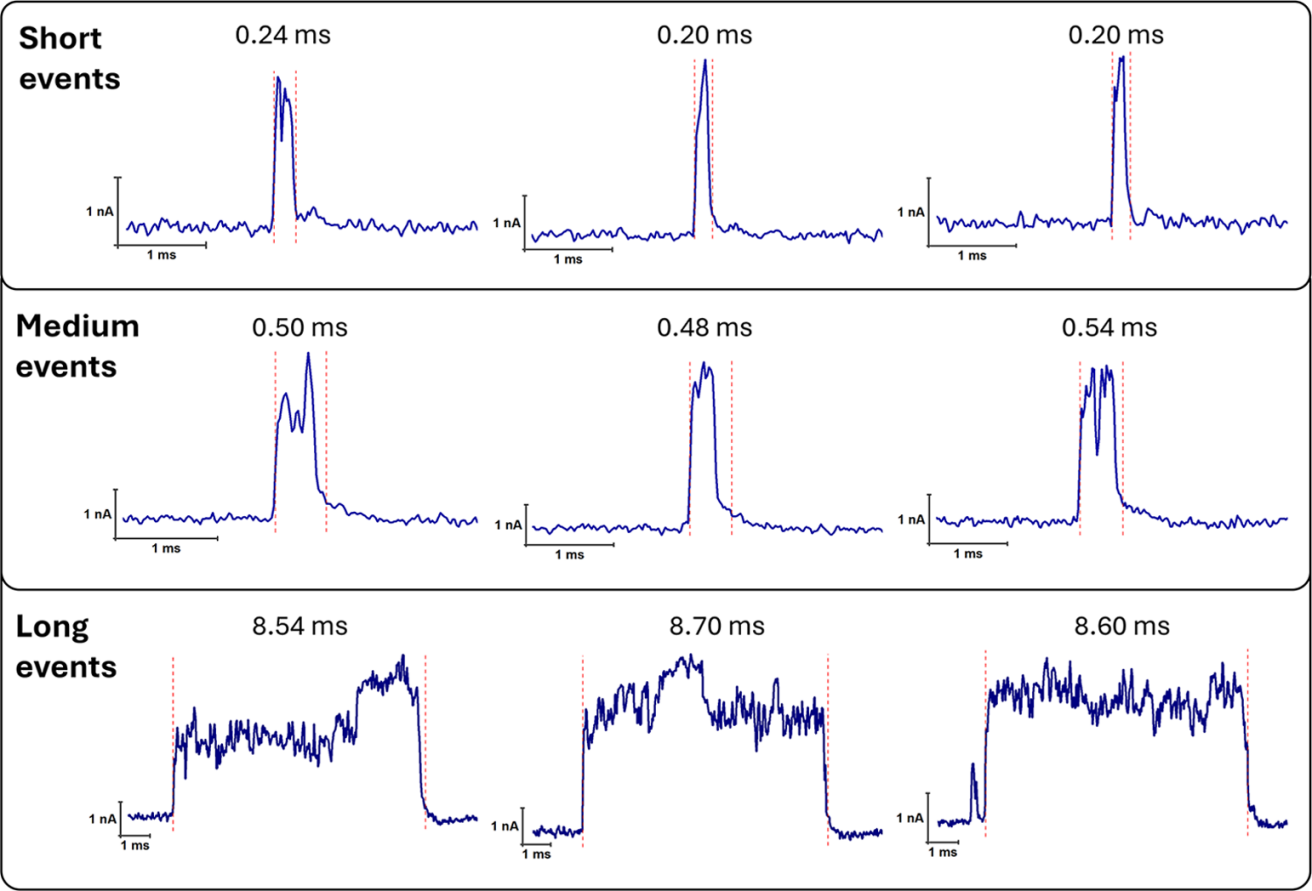
Representative nanopore current traces extracted using the developed application and classified based on dwell time: (Top) Short events (<0.3 ms), (Middle) Medium events (0.3–1.0 ms), and (Bottom) Long events (>1.0 ms). Short events potentially reflect small or minimally folded RCA products, medium events may correspond to moderately structured concatemers, and long events likely associated with highly folded or entangled RCA products.

To further strengthen these observations, additional nanopore measurements were performed with larger event counts across the three incubation times. Despite differences in absolute numbers, the proportional trends were highly consistent with our initial data set: short events dominated at 0.5 h but decreased progressively with longer incubation times, while long events increased steadily, reaching ∼26–30% at 2 h. Medium events remained relatively stable across all time points. This reproducibility across independent data sets reinforces the robustness of our classification approach and provides stronger statistical confidence that the observed changes in translocation signals reflect genuine, time-dependent structural evolution of RCA products.

In addition to dwell time-based categorization, two distinct complex translocation patterns were consistently observed (Figure 9). One potentially involved a sharp blockage followed by gradual signal recovery (Figure 9A), suggestive of the translocation of a dense structure translocating first, trailed by flexible segments. The other likely displayed a gradual increase in blockage leading into a peak event (Figure 9B), indicating the initial entry of a flexible structure followed by a denser, compact region. These signal features reflect the heterogeneous folding states of RCA products,^5,6,26^ which evolve during the amplification process and respond to the high ionic strength environment (3.6 M LiCl) used in the experiments.

**Figure 9:**
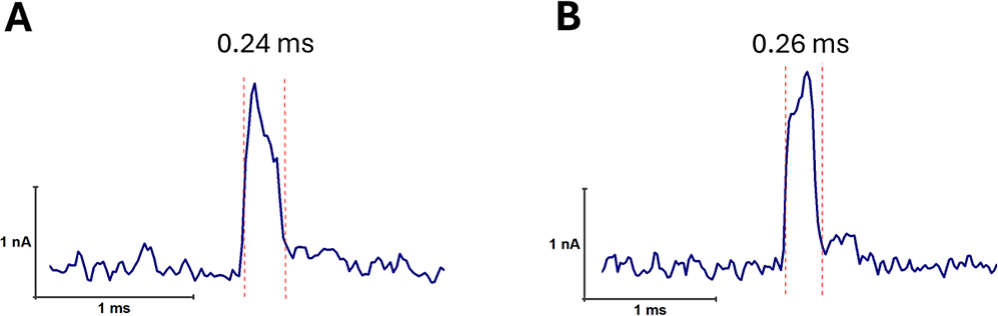
Examples of asymmetric nanopore translocation events observed in RCA products. (A) A high blockage event followed by a sloping recovery. (B) A sloping current trend leading into a high blockage. These features reflect variations in molecular structure and unfolding dynamics during translocation.

Together, dwell time classification and the identification of asymmetric or multistep translocation events offer molecular-level insight into RCA product elongation and folding. Longer dwell times reflect extended product lengths or stable folding, while irregular current traces indicate secondary structure formation and sequential unfolding. These features provide real-time, label-free evidence of structural heterogeneity in RCA products, thereby advancing our understanding of RCA kinetics and highlighting the power of solid-state nanopores for single-molecule structural profiling. To complement these single-molecule observations, we next performed atomic force microscopy (AFM) imaging to directly visualize RCA products at different time points.

### AFM Imaging of RCA Products

To complement nanopore translocation data and directly visualize RCA product morphology, AFM was performed on amplification samples collected at three incubation time points: 30 min, 1 h, and 2 h. Undiluted RCA reaction products (2 μL) were directly deposited onto freshly cleaved mica, allowed to adsorb briefly, and air-dried. Imaging was conducted in tapping mode under ambient conditions.

Based on standard RCA reaction conditions and yields, the amount of DNA deposited per sample is estimated to be in the tens to hundreds of nanograms, corresponding to a local mass concentration in the ng/μL to low μg/μL range—sufficient for effective surface coverage and visualization via AFM. Representative AFM images are shown in Figure 10A–C, revealing clear differences in the size and structural organization of the DNA products at each time point. At 30 min (Figure 10A), the RCA products appeared as compact and relatively short linear or coiled structures. These observations are consistent with nanopore data (Figure 6A,D), which exhibited short dwell times and lower % blockage currents, indicative of smaller and less folded molecules.

**Figure 10:**
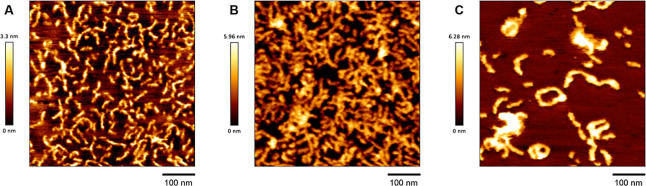
Atomic force microscopy (AFM) imaging of RCA products at different incubation times. Representative AFM images of rolling circle amplification (RCA) products collected after (A) 30 min, (B) 1 h (diluted sample shown; additional optimized images provided in Supporting Information Figure S3), and (C) 2 h of amplification. At 30 min, the RCA products appear as short, compact, and mostly linear or coiled structures. After 1 h, longer and more extended DNA strands are observed, with occasional branched and looped conformations. At 2 h, the RCA products display large, entangled, and highly heterogeneous morphologies. These time-dependent structural changes are consistent with nanopore sensing results (Figure 6), demonstrating increasing molecular length and complexity as amplification progresses. The AFM data provide complementary visual confirmation of RCA product growth and folding behavior during the reaction.

For the 1 h RCA sample, dilution of the product solution prior to deposition reduced aggregation and enabled clearer visualization of concatemeric DNA chains. As shown in Figure 10B and Supporting Information Figure S3, the RCA-1 h products exhibit elongated and partially branched structures, reflecting the extended but still intermediate amplification state. By 2 h (Figure 10C), RCA products displayed large, highly entangled, and occasionally aggregated structures, with a broad range of conformations observed. These AFM results correlate strongly with the nanopore data (Figure 6C,F), where translocation events exhibited significantly increased dwell times (up to >100 ms) and higher blockage levels, reflecting the presence of long, repetitive, and potentially self-folded DNA concatemers.

Importantly, although AFM and nanopore measurements were not conducted in identical buffer conditions—nanopore recordings utilized a high-salt environment (3.6 M LiCl), while AFM imaging was performed after depositing undiluted RCA samples onto mica followed by drying process—the consistent morphological and translocation trends observed across both platforms reinforce the robustness of the conclusions.

The AFM results provide physical and morphological confirmation of the structural evolution of RCA products over time, complementing the nanopore-based single-molecule sensing and Mfold-based secondary structure predictions. These combined data sets further validate the application of solid-state nanopores for real-time, label-free monitoring of RCA reaction kinetics and product formation at the single-molecule level.

## Conclusions

In conclusion, we have demonstrated a proof-of-concept integration of rolling circle amplification (RCA) with solid-state nanopore sensing for real-time, single-molecule monitoring of nucleic acid amplification. Using miR-21 as a model biomarker, our results reveal clear amplification-dependent changes in nanopore translocation signatures, characterized by progressively increasing dwell times, shifts in blockade currents, and the emergence of complex event types. These findings were consistently supported by PAGE validation, AFM imaging, and secondary structure predictions, together providing a coherent picture of RCA product growth and conformational diversity.

Our statistical event classification further confirmed the time-dependent shift in translocation behavior, with longer dwell times and increasing proportions of complex events observed at later amplification stages. Importantly, these trends were reproducible across independent data sets, reinforcing the robustness of the approach and increasing confidence in the observed time-dependent evolution of RCA products.

Although this study focused on three incubation times and a single nanopore size (∼8 nm), the consistency of the results establishes a solid foundation for future studies that expand to broader experimental conditions. Overall, these findings highlight the promise of solid-state nanopores as a powerful platform for label-free monitoring of nucleic acid amplification at the single-molecule level, with potential applications in molecular diagnostics and real-time biosensing.

## Supplementary Material



## Notes

Custom-developed nanopore event extraction web application (Figure S1); secondary structure prediction of circular P21 probe using Mfold (Figure S2); optimized AFM imaging of RCA-1 h products under diluted conditions (Figure S3); and Supplementary Note on estimation of final analyte and protein concentrations for nanopore experiments (PDF)

## Abbreviations

RCA: rolling circle amplification
miR-21: microRNA-21
AFM: atomic force microscopy
SiN: silicon nitride
T4 Rnl2: T4 RNA ligase 2
BSA: bovine serum albumin
dNTPs: deoxynucleotide triphosphates
HEPES: 4-(2-hydroxyethyl)-1-piperazineethanesulfonic acid
LiCl: lithium chloride
KCl: potassium chloride
NaCl: sodium chloride
NaClO: sodium hypochlorite
TBE: tris-borate-EDTA
PAGE: polyacrylamide gel electrophoresis.
