# Supplementary material for: Structural and Kinetic Profiling of Rolling Circle Amplification via Solid-State Nanopore Sensing Using miR-21 as a Model
Source: ACS Sens. 2025 Sep 16;10(9):7014–24. doi: 10.1021/acssensors.5c02039 (PMC12481570; doi:10.1021/acssensors.5c02039)
Supplement: Supplementary file 1 [file se5c02039_si_001.pdf]

Supporting Information

Structural and Kinetic Profiling of Rolling Circle Amplification via Solid-State Nanopore Sensing Using miR-21 as a Model

*Kawin Loha[1], Thitikorn Boonkoom[2], Harit Pitakjakpipop[2], Ibrar Alam[2], Alongkot Treetong[2], Poramin Boonbanjong[3], Itthi Chatnuntawech[2], Surat Teerapittayanon[2], Ulrich F. Keyser[4], Albert Schulte[1], and Deanpen Japrung[2]**

[1]School of Biomolecular Science and Engineering (BSE), Vidyasirimedhi Institute of Science and Technology (VISTEC), Rayong, 21210, Thailand.

[2]National Nanotechnology Center (NANOTEC), National Science and Technology Development Agency (NSTDA), Thailand Science Park, Pathumthani, 12120, Thailand.

[3] Program in Translational Medicine, Chakri Naruebodindra Medical Institute, Faculty of Medicine Ramathibodi Hospital, Mahidol University, Samut Prakan, 10540, Thailand.

[4]Cavendish Laboratory, University of Cambridge, Cambridge CB3 0HE, United Kingdom.

Application Development for Nanopore Event Extraction Web Application

A

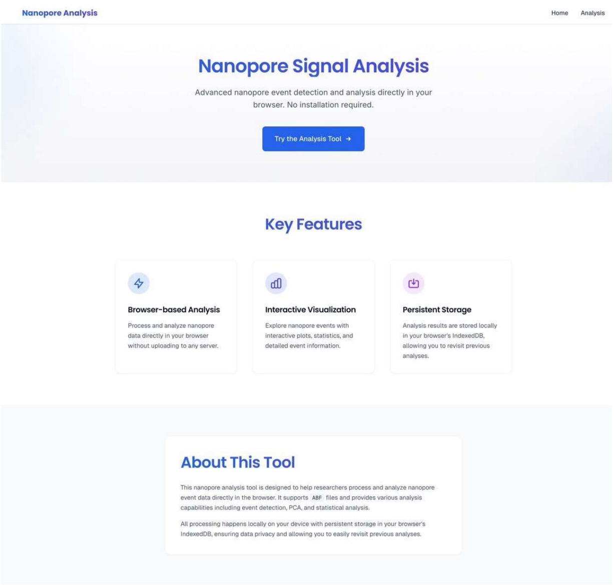

B

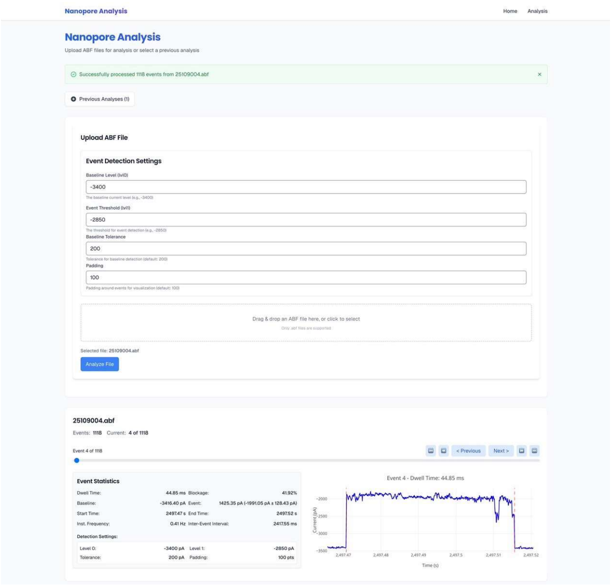

Figure S1. Graphical user interface (GUI) of the custom-developed nanopore signal analysis platform. (Left) Main homepage illustrating key features of the application, including browser-based analysis, interactive event visualization, and persistent data storage capabilities. (Right) Event detection interface where users can upload nanopore signal files, set customizable parameters such as baseline level (lvl0), event detection threshold (lvl1), baseline tolerance, and padding, and automatically extract translocation events. The output section displays event statistics and representative current traces and analysis of nanopore data.

Secondary Structure Prediction of Circular P21 Probe

The secondary structure of the circular padlock DNA probe (P21) was predicted using the Mfold web server [1] to simulate conformational behavior under experimental ionic conditions. The input sequence was configured as circular DNA, and folding calculations were performed at 25 °C with a sodium ion concentration of 3,000 mM to reflect the high-salt environment used in nanopore measurements. The output included multiple conformations ranked by Gibbs free energy (dG). Three representative structures with the lowest predicted free energies (+4.13, +4.99, and +5.06 kcal/mol) were selected for visualization and are shown in Supplementary **Figure S2.** The positive dG values suggest that secondary structure formation is thermodynamically unfavorable, supporting the interpretation that P21 remains largely unstructured and flexible in solution.

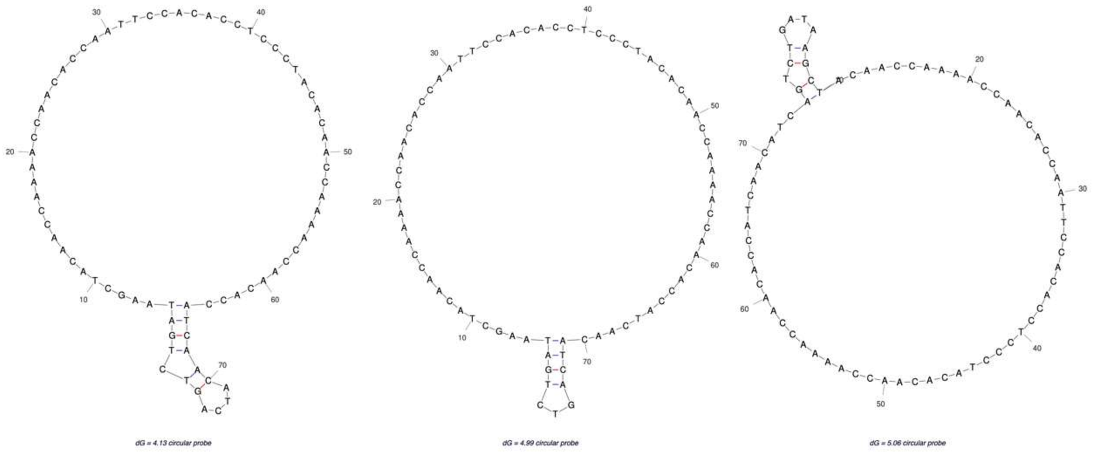

Figure S2. Predicted secondary structures of the circular P21 probe were generated using the Mfold web server under high-salt conditions (3 M Na^+, 25 °C), simulating the ionic strength used in nanopore experiments. The three structures, shown left to right, exhibit Gibbs free energy values of +4.13, +4.99, and +5.06 kcal/mol, respectively. The positive free energy values indicate that

secondary structure formation is not thermodynamically favored under these conditions. This supports the interpretation that the circular P21 probe remains largely unstructured and flexible in solution, consistent with the broad and variable translocation signals observed in nanopore sensing.

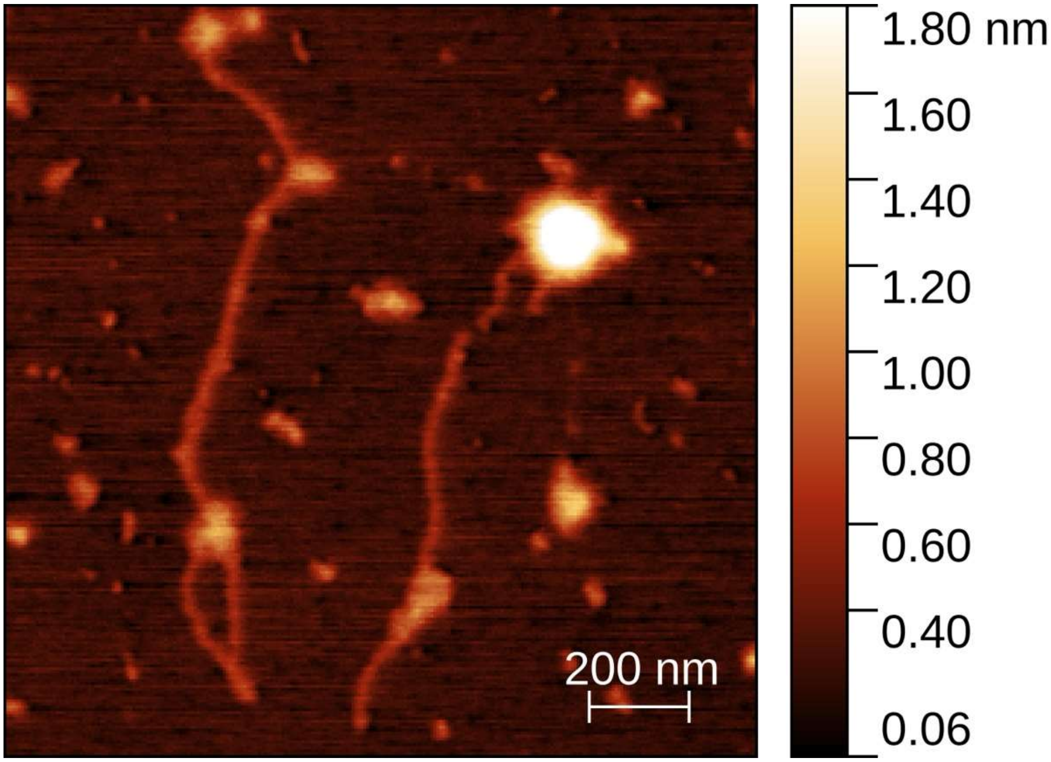

Figure S3. Optimized AFM imaging of RCA-1h products under diluted conditions. Atomic force microscopy (AFM) images of RCA products after 1 h of amplification, prepared under diluted sample conditions to minimize molecular aggregation and improve visualization of elongated concatemeric DNA chains. Compared to undiluted samples, the dilution step allowed clearer resolution of individual strands and reduced surface clustering, providing improved structural insight into the intermediate amplification stage. Scale bar: 200 nm.

Supplementary Note: Estimation of Final Concentrations for Nanopore Experiments

To evaluate the approximate concentrations of the ligation product and T4 RNA ligase 2 used in the nanopore sensing experiments, we calculated theoretical values based on input volumes, molecular weights, and dilution factors.

Ligation Product Concentration

The ligation reaction was performed in a total volume of 10 μL and contained:

1) 1 μL of 100 nM linear padlock probe (P21)
2) 1 μL of 10 nM miR-21
3) 1 μL of 10X ligation buffer
4) 1 μL (10 units) of T4 RNA ligase 2
5) 6 μL of RNase-free water

` Assuming complete ligation, the maximum theoretical concentration of the ligation product is limited by the lower concentration of the two hybridizing oligonucleotides (miR-21), i.e., 10 nM. After a 25-fold dilution into the nanopore buffer (3.6 M LiCl with 10 mM HEPES), the final estimated concentration of the ligation product is:

$$\frac{10\ \text{nM}}{25} = 0.4\ \text{nM}$$

This value represents the theoretical maximum under ideal conditions. Notably, 0.4 nM is within the detection range of solid-state nanopore sensors, which are capable of detecting individual molecular translocation events at sub-nanomolar levels under optimized conditions.

T4 RNA Ligase 2 Concentration

The ligation reaction included 10 Units of T4 RNA ligase 2 (1 μL), with a specific activity of approximately 100,000 U/mg. This corresponds to:

$$\frac{10\ \text{U}}{100{,}000\ \text{U/mg}} = 0.0001\ \text{mg} = 100\ \text{ng}$$

Assuming a molecular weight of ~65 kDa for T4 RNA ligase 2, this equates to:

$$\frac{100\ \text{ng}}{65{,}000\ \text{g/mol}} = 1.54\ \text{pmol}$$

After 25-fold dilution, the final concentration of T4 RNA ligase 2 becomes:

$$\frac{1.54\ \text{pmol}}{250\ \mu\text{L}} = 6.16\ \text{nM}$$

This low concentration, combined with the enzyme's near-neutral or slightly positive charge at pH 8, likely prevents electrophoretic entry into the nanopore under negative bias, consistent with the lack of detectable signal in the enzyme-only control condition.

Estimated Concentrations of Phi29 DNA Polymerase and BSA

To estimate the contribution of protein components to nanopore signals, we calculated the final concentrations of Phi29 DNA polymerase and bovine serum albumin (BSA) after dilution into the nanopore sensing buffer.

Phi29 DNA polymerase was added to the RCA reaction at 1 µL (10 U) in a 10 µL total reaction volume. Assuming a specific activity of 40 U/µg and a molecular weight of ~68 kDa, this corresponds to approximately 0.25 µg or 3.68 pmol of enzyme. Thus, the concentration in the RCA mix was ~368 nM. After a 25-fold dilution for nanopore analysis, the final concentration of Phi29 DNA polymerase was estimated to be approximately 14.7 nM.

BSA was present in the RCA reaction buffer at a typical concentration of 0.1 mg/mL (100 µg/mL), which equals 1.52 pmol/µL based on its molecular weight of ~66 kDa. In a 10 µL reaction, this results in 15.2 pmol of BSA or a concentration of 1.52 µM. Following a 25-fold dilution, the final BSA concentration in the nanopore detection solution was approximately 60.8 nM.

SI Reference

1. Mfold web server for nucleic acid folding and hybridization prediction. Nucleic Acids Research, 31(13), 3406–3415. https://doi.org/10.1093/nar/gkg595
